# Iron accumulation/overload and Alzheimer's disease risk factors in the precuneus region: A comprehensive narrative review

**DOI:** 10.1002/agm2.12363

**Published:** 2024-10-22

**Authors:** Sana Mohammadi, Sadegh Ghaderi, Farzad Fatehi

**Affiliations:** ^1^ Neuromuscular Research Center, Department of Neurology, Shariati Hospital Tehran University of Medical Sciences Tehran Iran; ^2^ Department of Neuroscience and Addiction Studies, School of Advanced Technologies in Medicine Tehran University of Medical Sciences Tehran Iran; ^3^ Neurology Department University Hospitals of Leicester NHS Trust Leicester UK

**Keywords:** Alzheimer's disease, iron, precuneus

## Abstract

Alzheimer's disease (AD) is a neurodegenerative disease that is characterized by amyloid plaques, neurofibrillary tangles, and neuronal loss. Early cerebral and body iron dysregulation and accumulation interact with AD pathology, particularly in the precuneus, a crucial functional hub in cognitive functions. Quantitative susceptibility mapping (QSM), a novel post‐processing approach, provides insights into tissue iron levels and cerebral oxygen metabolism and reveals abnormal iron accumulation early in AD. Increased iron deposition in the precuneus can lead to oxidative stress, neuroinflammation, and accelerated neurodegeneration. Metabolic disorders (diabetes, non‐alcoholic fatty liver disease (NAFLD), and obesity), genetic factors, and small vessel pathology contribute to abnormal iron accumulation in the precuneus. Therefore, in line with the growing body of literature in the precuneus region of patients with AD, QSM as a neuroimaging method could serve as a non‐invasive biomarker to track disease progression, complement other imaging modalities, and aid in early AD diagnosis and monitoring.

## Graphical abstract

Early Alzheimer's disrupts iron balance in the precuneus, a critical brain region for cognition. QSM, a post‐processing imaging technique, reveals this abnormal iron deposition, potentially offering a non‐invasive way to track disease progression and its link to metabolic, genetic, and vascular risk factors.

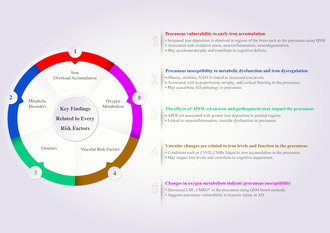

## 1 INTRODUCTION

Alzheimer's disease (AD) is an age‐related neurodegenerative disease (NDD) with cognitive, behavioral, and psychological symptoms that ultimately lead to cognitive decline and memory loss.^1^, ^2^ It is the most common form of dementia,^3^ contributing to 60%–70% of cases,^4^ and is the fifth‐leading cause of death among Americans aged 65 and older.^5^, ^6^ Currently, more than 55 million people have dementia worldwide, with more than 60% living in low‐income and middle‐income countries.^7^ This number is projected to increase to 152 million by 2050.^8^ The global number of patients with AD dementia, prodromal AD, and preclinical AD is estimated to be 416 million, or 22% of all persons 50 and above.^9^ Recent advances in molecular biology and neuroimaging have highlighted the multifactorial interplay between genetic, environmental, and metabolic factors that contributes to AD pathogenesis.^3^, ^10^, ^11^, ^12^ Although aging is the strongest risk factor,^13^, ^14^, ^15^, ^16^ accumulating evidence suggests that metabolic dysfunction and iron dysregulation also significantly influence AD pathogenesis.^3^, ^17^, ^18^, ^19^, ^20^

Iron bound to hemoglobin plays a crucial role in oxygen transport and protein functions.^21^ They also play roles in DNA synthesis, repair, and iron homeostasis.^22^ Iron plays a role in myelination, antioxidant enzyme function, and neurotransmitter synthesis.^22^ Factors such as aging, inflammation, metal dysregulation, and oxidative stress disrupt iron metabolism.^22^, ^23^ Iron overload in the body, which is often associated with conditions such as hereditary hemochromatosis or excessive iron intake, can cause a variety of problems such as oxidative stress, inflammation, and damage to cells and neurons.^24^, ^25^ One aspect of AD that has received increasing attention is the role of iron accumulation and dysregulation of iron homeostasis in the brain.^26^ Several studies have used magnetic resonance imaging (MRI) to observe changes in magnetic susceptibility (*χ*) changes, particularly to detect increased iron content using T2* mapping, T2* gradient echo imaging, quantitative T2* and transverse relaxation rates R2 and susceptibility‐weighted imaging (SWI).^3^, ^27^, ^28^ Magnetic susceptibility is a physical feature that aids the quantification of specific materials and chemical identification.^29^ However, these techniques suffer from blooming artifacts and cannot quantify the tissue susceptibility.^30^ Quantitative susceptibility mapping (QSM) is an MRI method that enables the measurement of materials that cause changes in susceptibility.^31^, ^32^

It is challenging to explain AD using a single pathological pathway. Aβ triggers a chain of events in AD including tau pathology.^22^ Oxidative stress, neural inflammation, autophagy, apoptosis, and metal dysregulation have also been observed in AD.^3^, ^22^ Metal dyshomeostasis, intracellular iron deposition, and ferroptosis are key causes of neuronal loss in patients with AD.^22^, ^33^ In AD, iron overload can result in increased production of Aβ by altering the expression and processing of amyloid precursor protein (APP).^34^ In the early stages of AD, amyloids tend to accumulate mainly in the temporal, basal frontal, and occipital lobes. As the disease advances, amyloid deposition spreads to other areas of the brain.^2^, ^35^ Positron emission tomography (PET) amyloid images show a characteristic distribution in different regions of the brain, beginning in the precuneus, inferior temporal gyrus, orbitofrontal cortex, and posterior cingulate gyrus.^36^, ^37^ Iron is found at high levels in plaques and can cause Aβ to aggregate, which is cytotoxic in vitro.^34^, ^38^ Iron overload can also lead to autophagic cell death and ferroptosis as well as contribute to the phosphorylation of tau protein, leading to the formation of neurofibrillary tangles (NFTs), all of which increase the risk of NDDs.^33^, ^34^

Interestingly, previous advanced non‐invasive MRI studies have consistently reported emerging evidence of iron accumulation,^30^, ^39^, ^40^ hypoperfusion,^40^, ^41^, ^42^, ^43^, ^44^, ^45^, ^46^, ^47^, ^48^, ^49^, ^50^, ^51^, ^52^ atrophy,^39^, ^42^, ^44^, ^51^, ^53^, ^54^, ^55^, ^56^, ^57^, ^58^, ^59^, ^60^, ^61^, ^62^, ^63^, ^64^, ^65^, ^66^, ^67^, ^68^, ^69^, ^70^ cortical thinning,^54^, ^61^, ^66^, ^71^, ^72^, ^73^ and connectivity disruptions^74^, ^75^, ^76^, ^77^, ^78^, ^79^, ^80^, ^81^ in the precuneus of patients with AD. Remarkably, the precuneus represents a central functional hub affected by AD.^80^

**FIGURE 1:**
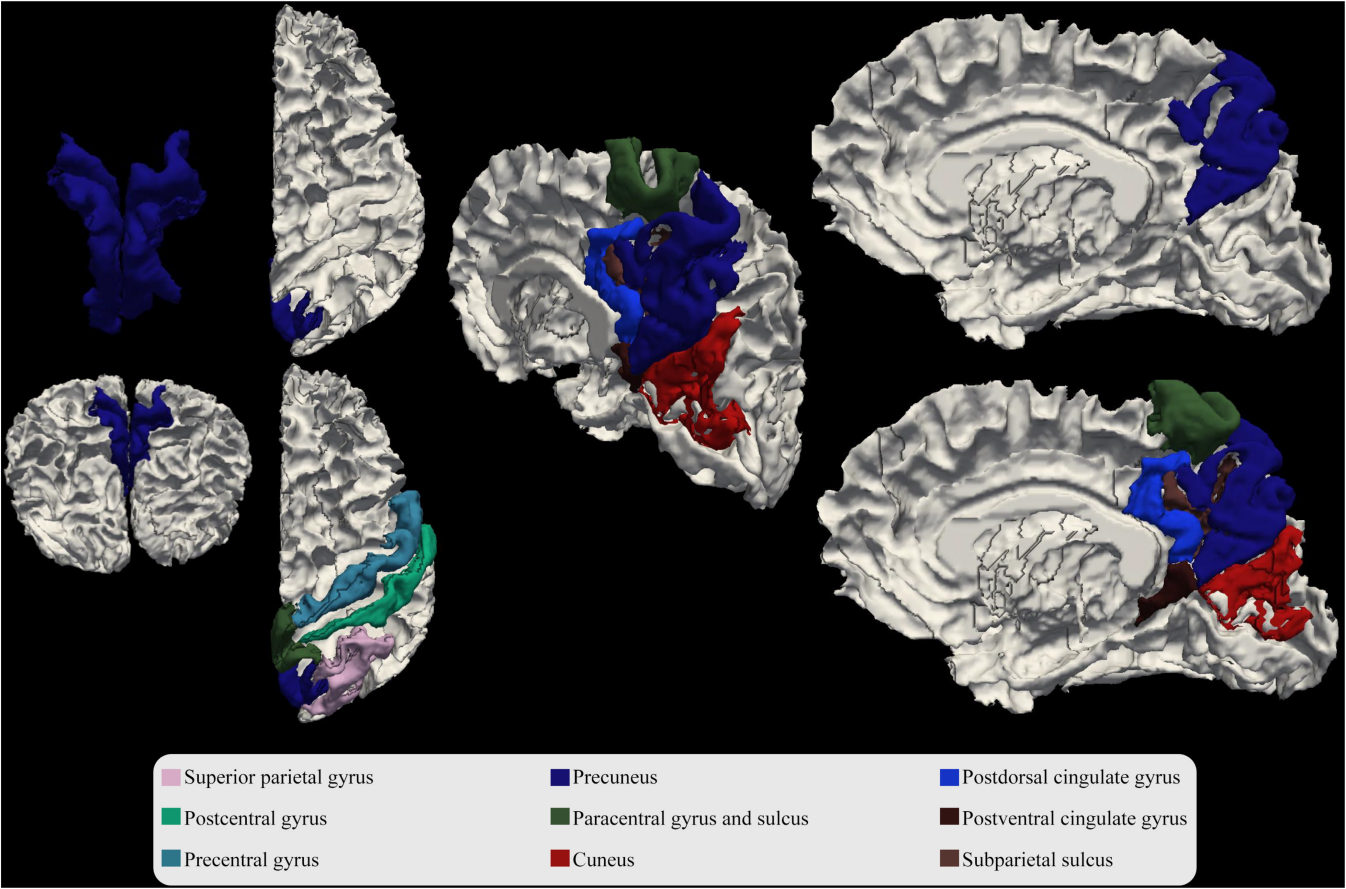
The precuneus and associated regions.

The precuneus, a part of the posteromedial parietal lobule, is closely related to the posterior parietal cortex in front of the cuneus and is limited by the cingulate sulcus^82^ (Figure 1). Early brain atrophy and functional changes in the precuneus region are considered vulnerable areas during the transition from mild cognitive impairment (MCI) to dementia and AD.^83^, ^84^, ^85^, ^86^ This region is involved in a range of cognitive functions, including self‐referential processing, visuospatial attention, navigation, and autobiographical and episodic memory.^87^, ^88^ Iron deposition in the precuneus has been associated with cognitive decline, gray matter (GM) atrophy, and impaired functional connectivity (FC), suggesting its potential role in disease progression.^30^, ^39^, ^40^ The precuneus experiences early amyloid deposition, NFT formation, vascular dysfunction, and cerebral hypoperfusion, which are believed to initiate a neurodegenerative cascade that leads to cognitive decline and early AD symptoms.^40^, ^89^, ^90^, ^91^, ^92^

Another significant contributor to the progression of AD is obesity, particularly obesity in midlife, along with associated metabolic and long‐term conditions, such as type 2 diabetes (T2DM) and non‐alcoholic fatty liver disease (NAFLD).^93^, ^94^, ^95^ These metabolic syndromes have become epidemics in recent years.^96^, ^97^, ^98^, ^99^ These conditions independently increase AD risk by systemically promoting insulin resistance (IR), adipocytokine dysregulation, chronic inflammation, and increased iron deposition.^17^, ^100^, ^101^, ^102^ Given the role of adiposity in altering body iron storage^103^, ^104^ and the implication of abnormal iron levels in AD neuropathology,^17^, ^105^ QSM may provide insights into the interactions between obesity‐linked risk and AD pathogenesis. Therefore, there is a significant overlap between the vulnerability of the precuneus to amyloid deposition and dysregulation in obesity and other associated metabolic functions,^54^, ^106^ more research is needed to understand the interrelationships between these factors.

Evidence suggests that early detection and intervention for MCI, particularly amnestic MCI (aMCI), may be associated with a reduced prevalence of mild Alzheimer's symptoms and facilitate earlier diagnosis of the disease.^107^ Therefore, it is critical to understand the early changes that occur in the brain, particularly in the precuneus/posterior parietal cortex, due to iron deposition/accumulation, alterations in other MR neuroimaging biomarkers, and metabolic disorders. This literature review aimed to synthesize current knowledge on the association between iron overload, iron accumulation, and metabolic disorders in the context of AD pathophysiology in the precuneus. In addition, we introduce QSM (*χ*) as a potential emerging neuroimaging biomarker and explore its potential utility in predicting the onset and progression of AD.

## 2 PATHOPHYSIOLOGY OF ALZHEIMER'S DISEASE

The key features of AD include the deposition of β‐amyloid (Aβ) plaques and hyperphosphorylated tau tangles, as well as neuronal loss and glial activation.^3^ Although research has historically focused on Aβ pathology, recent evidence suggests that tau pathology may be more closely associated with the onset and progression of AD symptoms.^108^, ^109^ However, the relationship between Aβ and tau pathology remains controversial.^110^, ^111^ Reactive oxygen species (ROS)‐induced oxidative damage is associated with NDDs, diabetes, and aging.^112^, ^113^, ^114^ Elevated ROS levels contribute to AD progression, neurodegeneration, Aβ plaque deposition, and proteinopathy in the brain.^45^, ^115^ ROS can alter the expression and function of iron‐regulatory proteins, such as ferroportin and ferritin, which store and transport iron within cells. ROS can also affect the balance between iron and other metals in the brain, leading to iron deposition in the brain. Nevertheless, iron accumulation and deposition have drawn the interest of researchers as potential new neuroimaging biomarkers that reflect the early diagnosis and disease severity in AD.^3^, ^116^ Changes in iron metabolism and accumulation have been detected in the brain tissues of patients with AD, with iron found in the same locations as senile plaques and NFTs.^117^, ^118^ Abnormal proteins in AD brain tissues bind ferric iron and convert it to ferrous iron, which leads to the generation of harmful hydroxyl radicals, contributing to the ferroptosis pathway.^119^ As a result, abnormal iron accumulation is suggested to be involved in the development of AD because it is present in the initial phases of the disease, interacts with Aβ and tau, and plays a significant role in inflammation associated with AD.^118^

Many brain regions affected by AD show early signs of atrophy without prominent deficits in neuroimaging and metabolism biomarkers.^51^, ^70^ These findings offer unique insights into AD pathophysiology. However, several neuroimaging^54^, ^56^, ^120^, ^121^, ^122^, ^123^, ^124^, ^125^ and neuropsychological^124^, ^126^, ^127^ studies have reported brain structural alterations and cognitive domains impairments such as executive functions and episodic memory in obese individuals.

### 2.1 Obesity and adipose tissue changes

Obesity, which affects 30% of the world's population (2.1 billion people), is caused by genetic, behavioral, and environmental factors.^128^ Obesity is associated with NDDs, such as AD, multiple sclerosis (MS), and Parkinson's disease (PD).^129^, ^130^ Different types of adipose tissue, including brown adipose tissue (BAT), white adipose tissue (WAT), subcutaneous adipose tissue (SAT), and visceral adipose tissue (VAT), have distinct roles in energy metabolism and adipocytokine secretion.^130^ The VAT and SAT are associated with obesity and metabolic disorders.^131^ BAT and WAT are involved in energy metabolism.^132^ BAT is linked to reduced VAT levels and increased SAT levels, leading to a decrease in central obesity.^133^ Increased VAT has been potentially associated with accelerated brain aging, decreased cortical thickness (CTh), cerebral atrophy, and lower cognitive scores.^134^ Obesity in midlife is a risk factor for developing AD, regardless of other metabolic factors, such as diabetes and hypertension.^135^ However, high BMI/body weight in late life is protective against AD and cognitive decline.^135^, ^136^ Additionally, BMI, waist‐to‐hip ratio (WHR), and body fat percentage (BFP) have been studied to assess their relationships with brain structure and function.^123^ These obesity‐related measures have been linked to GM atrophy, particularly in regions sensitive to AD pathology, such as the precuneus and posterior cingulate cortex (PCC).^54^, ^123^

In a recent study, Chen et al.^123^ utilized MRI data and a Mendelian randomization method to establish a direct link between obesity and cerebral structure. Their findings revealed that the genetically predicted VAT was connected to the surface area of the isthmus cingulate and precuneus. Additionally, genetically predicted BFP was linked to a reduced cortical surface area in the precuneus. The results of the multivariable Mendelian randomization method indicated that a higher BFP was associated with reduced thickness of the fusiform gyrus and a smaller surface area of the precuneus. They also discovered a strong relationship between obesity, brain abnormalities, and neuropsychiatric diseases such as AD.

A study by Dolatshahi et al.^54^ found that the ratio of visceral to VAT/SAT was significantly associated with the Pittsburgh compound B (PiB) standardized uptake value ratio (SUVRs) in the right precuneus cortex, particularly in males. This association was linked to lower CTh in areas associated with AD, such as the bilateral temporal cortices and parahippocampal and cingulate cortices. Additionally, higher BMI and IR were associated with a lower CTh in the temporal pole. In midlife, cognitively normal adults, higher visceral obesity, and IR were associated with higher amyloid pathology in the right precuneus cortex and lower CTh in the areas associated with AD.

On the other hand, obesity is characterized by chronic, low‐grade, systemic inflammation that is associated with anemia in chronic disease and can alter iron metabolism parameters.^137^ Changes in iron metabolism parameters are common in overweight and obese individuals, particularly during adolescence and morbid obesity.^138^ These alterations include increased ferritin, hepcidin, and hepatic iron content^139^ and decreased hemoglobin, transferrin, and total iron binding capacity (TIBC).^137^ Serum ferritin levels have been associated with obesity indicators.^140^, ^141^ Impaired functional iron status is linked to inflammation in adipose tissue and increased hepcidin expression.^138^ Hepcidin is a hormone that regulates iron absorption from the gut and iron release from macrophages. Increased hepcidin production in the brain can lead to decreased iron release from macrophages, which can contribute to brain iron deposition.

Over the years, obesity has been recognized not only as an independent risk factor for AD but also as a condition that exacerbates existing neuropathological changes.^142^ However, the impact of obesity on brain iron levels and related outcomes such as neurodegenerative processes has not been thoroughly investigated.^143^, ^144^ Thus, obesity‐related iron accumulation in the brain is a newly emerging research area that is gaining attention for its potential connection to the development and progression of NDDs such as AD.

In 2019, a preliminary study examined the effects of diet‐induced obesity on iron levels in the brain, F2‐isoprostane concentration (a marker of oxidative stress), and α‐synuclein expression in specific brain regions. The study revealed that obesity led to significant variations in iron levels in the midbrain and thalamus, but not in the hippocampus or striatum when compared with the control group of mice.^143^ Additionally, levels of neurodegenerative markers are increased in the midbrain. This study confirms that brain iron metabolism responds regionally to environmental stress, suggesting that obesity‐induced changes may be linked to neurodegeneration.

Excess iron can lead to dysfunction in adipose tissue and IR.^145^ Studies have shown that markers of iron status in blood are linked to obesity and adipose tissue.^144^ In another preliminary study, ferritin levels were positively correlated with both VAT and SAT and negatively correlated with hepatic fat content.^146^ In line with this study, a study was conducted on 131 healthy subjects with and without obesity to evaluate the connection between iron status and changes in abdominal adipose tissue over 1 year.^140^ The findings indicated that higher levels of serum hepcidin and ferritin were linked to increased VAT and SAT, whereas transferrin and TIBC were inversely associated with these changes. These connections were predominantly observed in women and non‐obese individuals and were not influenced by insulin sensitivity. This study indicated that serum hepcidin levels are associated with longitudinal changes in abdominal adipose tissue independent of insulin sensitivity. It is worth noting that adipocytes express high levels of transferrin, a key protein that binds iron in the blood, which is essential for adipocyte function and insulin sensitivity.^147^

Moreover, recent research has revealed a connection between serum ferritin and increased IR in adipose tissue (adipose‐IR) as well as a higher lipid accumulation product (LAP) and visceral adiposity index (VAI).^148^ This indicates that ferritin may serve as an early indicator of the potential development of adipose tissue dysfunction in obese.^148^ Furthermore, increased serum ferritin and iron levels have been linked to decreased markers of adipocyte differentiation, and reducing iron levels through phlebotomy improves glucose tolerance.^149^ This suggests changes in iron storage and chronic inflammation in individuals with obesity.

High body iron stores have been shown to affect fat growth in animals, hindering the formation of fat cells and the action of insulin.^140^, ^150^ Mice with obesity and diabetes had increased accumulation of iron in their fat tissue, which was linked to impaired fat cell development.^140^, ^150^, ^151^

At the same time, although all these observations indicate a decrease in the GMV and/or a decrease in the CTh in different regions, such as the precuneus, the exact relationship between brain structure and obesity remains unclear.^123^ Hence, there is a need for additional early neuroimaging biomarkers such as QSM.

### 2.2 Metabolic and chronic disorders, and related to the risk of AD

Iron is widely acknowledged to have a significant effect on the phenotypic expression of metabolic disorders.^140^, ^146^ In a human study, increased MRI markers of iron accumulation in adipose tissue were associated with a range of disorders such as obesity, IR, and adipose tissue dysfunction.^147^ Furthermore, a recent study showed that low levels of iron in adipose tissue can restrict the absorption of intestinal fat and reduce the intake of calories in mice fed a high‐fat diet, highlighting the significance of adipose tissue iron in overall metabolism.^152^ Thus, investigating the relationship between iron metabolism and temporal changes in obesity, IR, adipose tissue alteration, and other related metabolic and chronic disorders could help establish these indicators as predictors of body composition changes related to AD pathophysiology.

### 2.3 Leptin

In a previous study, the association between leptin and AD was explored.^135^ Leptin, an adipocytokine primarily secreted by the adipose tissue, has been implicated in the regulation of appetite, energy expenditure, cognitive function, and inflammatory mechanisms associated with obesity.^135^, ^153^ Adipose tissues, including the VAT and SAT, release various adipokines including leptin, which may disrupt iron homeostasis in the brain and contribute to neuroinflammation.^154^ In particular, VAT contributes to inflammation and IR, thereby affecting the cardiovascular health.^155^ Additionally, adipokines released by VAT are associated with systemic inflammation and IR.^156^ Specifically, higher amounts of VAT in midlife are linked to Alzheimer's development.^54^, ^157^ The potential impact of adipose tissue on neurodegenerative conditions such as AD is also under investigation.^158^

### 2.4 Insulin resistance and diabetes

It is well established that obesity leads to systemic metabolic dysregulation, including IR and altered adipose tissue function.^159^ IR occurs when there is an abnormal biological reaction to insulin stimulation, leading to disruption of various molecular pathways in the body's target tissues.^160^, ^161^ It has been suggested that IR may contribute to the pathogenesis of AD by influencing the clearance of Aβ peptides and the phosphorylation of tau protein.^130^, ^162^

Iron storage and adipose tissue inflammation are associated with IR and T2DM.^163^ Insulin regulates glucose metabolism and has various roles in the brain, such as regulating the metabolism of neurons and glial cells, managing glucose levels, and influencing cognitive functions.^164^ IR is a risk factor for neurodegeneration and cognitive impairment, and neuroimaging can reveal cerebral abnormalities associated with IR, including alterations in white matter (WM) microstructure, neural activity, brain volume, and brain metabolism over time.^164^, ^165^, ^166^ Brain regions associated with IR pathophysiology are more prominent in the dorsal nervous system, such as the precuneus, cingulate cortex, occipital lobe, and WM tracts.^164^ These structures are vulnerable to AD pathology and play a critical role in cognitive processes such as memory and executive functioning.^167^ Patients with metabolic disorders, such as T2DM and obesity, exhibit higher variability in their results than asymptomatic individuals do. This could be attributed to the interplay between multifactorial components.^164^ Overall, neuroimaging biomarkers are highly advantageous for detecting initial cerebral structure and functional abnormalities in brains with IR. These biomarkers provide crucial evidence for understanding the underlying neuronal changes involved in cognitive decline in IR.

### 2.5 Non‐alcoholic fatty liver disease

NAFLD is closely linked to obesity and can begin in childhood.^168^ Research indicates that liver diseases, even in the early stages, may be associated with brain aging.^169^ Changes in serum iron levels are common in adult NAFLD patients, leading to dysmetabolic iron overload syndrome.^168^ Interest in the impact of NAFLD on brain health is growing due to shared risk factors with cardiovascular disease, diabetes, and obesity, as well as common underlying mechanisms such as inflammation and endothelial dysfunction.^169^ NAFLD has been found to have an independent association with reduced brain activity and cognitive impairment, regardless of other concurrent health conditions.^169^ Given the high prevalence of NAFLD, interventions targeting it, such as lifestyle changes and weight reduction, could help to prevent cognitive impairment.

A study using MRI to examine liver steatosis in male adolescents found that ferritin was associated with liver fat and VAT.^168^ The study found that ferritin levels in obese male adolescents are primarily affected by liver fat and inflammation rather than body iron status. However, no association was observed between transferrin and soluble transferrin receptors, with ferritin levels of.^168^ Adiposity has been linked to cognitive impairment, AD, and related disorders (ADRD).^134^, ^170^ BMI is commonly used to measure adiposity, but it may not accurately represent the regional fat distribution.^171^, ^172^ Studies suggest that cognitive impairment and dementia, specifically AD, are more likely to occur in individuals with varying fat deposits and regional fat distributions.^134^ However, the cognitive outcomes associated with different fat deposits can vary, and these deposits can impact the brain in distinct ways.

In another study, a higher R2* value in adipose tissue, possibly indicating iron content, was correlated with liver iron stores in obese individuals.^173^ Additionally, VAT iron levels appear to be linked to serum cholesterol levels in individuals with obesity. It is important to mention that, while VAT and NAFLD have been studied, they could be potential metabolic biomarkers for future studies on the accumulation of iron in the brain. This could include investigating hallmark anatomical regions, such as the precuneus, and their relationship with the risk of AD and related disorders. Overall, brain iron deposition can be increased in NAFLD due to the increased expression and function of hepcidin, IR, and oxidative stress.

### 2.6 Vascular risk factors

A significant risk factor for dementia and cognitive impairment is cerebral small vessel disease (CSVD).^174^ Cerebral microbleeds (CMBs) are frequently observed in individuals aged 45 years and above, with a prevalence rate of 5%–35%.^175^ Tuladhar et al.^176^ have identified specific brain areas, including the superior frontal gyrus, precuneus, superior occipital gyrus, thalamus, and putamen as crucial nodes of CSVD. Recent research has shown that iron accumulation in the precuneus is an independent risk factor for MCI in patients with CMBs.^177^ This study found that the susceptibility values in the precuneus differed significantly between patients with CMBs and MCI and those without MCI. The accumulation of iron in distant rich‐club nodes owing to CMBs may be responsible for this difference. Logistic regression analysis revealed that only higher susceptibility values in the precuneus were associated with cognitive decline in patients with CMBs.^177^ Additionally, in a comparative study involving patients with PD, those with MCI showed a higher level of vulnerability in the precuneus region, as indicated by voxel‐wise QSM results. This suggests that the precuneus may be a potential target area for future MCI research in PD patients.^178^

### 2.7 Genetics risk factors

Cerebral autosomal dominant arteriopathy with subcortical infarcts and leukoencephalopathy (CADASIL) is a rare hereditary condition that affects the small blood vessels in the brain.^179^ Mutations in neurogenic locus notch homolog protein 3 (NOTCH3) are responsible for this condition, which poses a high risk of dementia and stroke in middle‐aged adults.^180^ CADASIL can lead to cognitive, mood, and behavioral disruptions.^181^ Dementia is usually diagnosed in the late stages, and cognitive impairment is uncommon, but can occur without significant vascular events.^182^, ^183^ Early onset dementia, which causes cognitive decline before the age of 65 years, can result from various factors such as familial AD (FAD) and metal metabolism disorders.^182^

Conventional MRI shows features similar to those of CSVD and CMBs.^2^, ^182^ The most common MRI finding associated with CADASIL is hyperintensity in the basal ganglia and WM in T2‐w, primarily in the periventricular and parietal regions.^184^, ^185^ Two recent studies have provided insights into SVD pathology in FAD and the genetic complexity of AD and related dementia.^186^, ^187^ The first study revealed Aβ‐independent SVD pathology in presenilin 1 (PSEN1) FAD, which is similar to CADASIL but milder on MRI.^187^ A second study identified a pathogenic NOTCH3 variant that causes CADASIL‐like features in an AD family, highlighting the genetic complexity of AD.^186^ These findings underscore the interaction between genetic and vascular factors in understanding the clinical and pathological spectrum of AD and its related disorders.

On the other hand, iron accumulation in the brain is suggested as a pathophysiological mechanism in CADASIL, especially in deep GM.^179^, ^188^, ^189^ This susceptibility signal in the putamen and caudate may serve as a biomarker of CADASIL severity.^179^, ^188^ Additionally, iron deposition in the basal ganglia is associated with cognitive dysfunction.^179^, ^190^ Therefore, iron accumulation and vascular changes in the brain may be potential factors in CADASIL, with studies suggesting that they may be linked to the severity of clinical disease and WM hyperintensity.^179^, ^188^

Kagerer et al.^191^ identified a synergistic association between elevated magnetic susceptibility and the presence of the APOE‐ε4 gene, resulting in heightened default mode network (DMN) activity, particularly in the precuneus, PCC, and lateral parietal cortex. Another study by van Bergen et al.^192^ observed that APOE‐ε4 carriers within the MCI group displayed significantly higher susceptibility in the precuneus and parietal regions than individuals without APOE‐ε4. Another recent study included 24 individuals who were not carriers of APOE‐ε4, 22 heterozygotes, and 20 homozygotes in the initial stages of AD. The study discovered that an increased APOE‐ε4 dose was linked to a reduction in the effective clearance of brain waste, such as Aβ and iron, through the blood–brain barrier.^193^

Studies have found associations between APOE‐ε4 and heightened DMN activity, increased magnetic susceptibility in the precuneus and parietal regions, and decreased brain waste clearance. This evidence suggests that the presence of the APOE‐ε4 allele affects multiple mechanisms implicated in AD pathogenesis including neuroinflammation, vascular dysfunction, and amyloid beta accumulation.

### 2.8 Atypical AD evidence

Limited knowledge exists regarding the distribution of iron in atypical clinical presentations of AD, such as logopenic progressive aphasia (LPA) and posterior cortical atrophy (PCA). LPA is characterized by language deficits, including difficulty with word retrieval, repeating sentences, and phonological errors, while PCA involves visuospatial and visuoperceptual deficits.^39^, ^194^, ^195^ Moderate evidence indicates greater precuneus susceptibility in both PCA and LPA. Strong evidence also suggests greater susceptibility in the precuneus in PCA than in typical AD (tAD).^39^ PCA and LPA exhibited a higher susceptibility than tAD in the precuneus. This observation is consistent with previous research that links precuneus atrophy with the deterioration of autobiographical memory in PCA.^39^, ^67^, ^196^, ^197^ Another study revealed a connection between perceptual specifics of personal memory and GM density in the precuneus, located on the right side of the brain.^67^ This suggests a correlation between certain areas of the brain and PCA (specific atrophy),^67^ and an acceleration of this process may occur due to the deposition of iron.^198^, ^199^

However, further research is needed to determine whether increased iron deposition accelerates atrophy in the precuneus and contributes to deficits in language, memory, and visuospatial abilities observed in these AD variants.

## 3 IRON OVERLOAD/ACCUMULATION AND INCREASE RISK OF AD

Iron accumulation in the brain is frequently observed in different types of NDDs.^200^ Histochemical and imaging studies have found iron accumulation in brain regions with histopathological alterations in AD patients, along with associations with Aβ aggregates, NFTs, and activated microglia.^3^, ^109^, ^117^, ^118^, ^199^, ^201^, ^202^, ^203^, ^204^, ^205^, ^206^, ^207^, ^208^, ^209^, ^210^, ^211^, ^212^, ^213^, ^214^ Strong imaging evidence indicates that dysregulation of iron in the neuronal system and alterations in susceptibility could be significant hallmarks of AD development.^25^, ^27^, ^30^, ^39^, ^40^, ^192^, ^202^, ^215^, ^216^, ^217^, ^218^, ^219^, ^220^, ^221^, ^222^ Iron has been found to be associated with amyloid plaques in conventional studies using ex vivo and in vivo mouse models. This was determined by analyzing the T2* properties and creating maps of the iron distribution.^38^, ^118^, ^223^, ^224^, ^225^ Moreover, the loss of myelin in WM leads to an increase in susceptibility, bringing it closer to that of GM.^226^, ^227^ Demyelination and accumulation of iron contribute to increased susceptibility of local tissue susceptibility.^27^ Therefore, a potential risk factor for AD is believed to be high levels of iron in the brain.

Excessive iron accumulation in the brain can initiate a chemical reaction called the Fenton reaction, leading to increased oxidative stress, which is a hallmark of AD.^228^ This oxidative stress can activate programmed cell death pathways in neurons, causing damage to crucial proteins and lipids, ultimately resulting in synaptic dysfunction and the death of neuronal cells.^34^, ^229^, ^230^ Current research highlights the significance of iron in the regulation of tau phosphorylation and aggregation, which in turn may contribute to the formation of neurofibrillary tangles in NDDs such as AD.^34^, ^231^, ^232^ There appears to be a significant interaction between iron and tau, which influences disease progression and symptoms.^34^, ^231^ Interestingly, tau also binds to iron, leading to its aggregation and possible deposition as iron‐rich tangles in AD brains.^231^ Moreover, elevated iron levels can increase tau phosphorylation in cultured neurons, suggesting a potential link between iron elevation and pathological tau in AD.^34^, ^202^, ^231^ The findings of decreased total tau levels in the cortex of AD patients and the requirement of tau for the trafficking of APP to the neuronal membrane support the idea that disruptions in tau and APP levels can lead to the retention of iron in neurons, which is a hallmark of AD.^34^, ^233^

Ferroptosis is a form of the cell death pathway that is not related to apoptosis and is dependent on iron, which has noteworthy implications in NDDs.^234^ Although they are distinct from apoptosis and autophagy, they share common protein molecules.^234^, ^235^ Current research has focused on understanding ferroptosis and its interplay with these pathways, thereby providing insights into its mechanisms and potential therapeutic approaches.^234^, ^236^ Ferroptosis occurs due to failure in the clearance of lipid peroxides, the presence of redox‐active iron, and oxidation of phospholipids containing polyunsaturated fatty acids (PUFAs).^234^ The regulation of oxidative stress and inflammatory responses is controlled by iron metabolism and lipid peroxidation signaling, which are the central mediators of ferroptosis. In addition, elevated iron levels can induce ferroptosis independently of free radical toxicity, holding therapeutic promise through iron chelation or uptake inhibition.^140^, ^234^, ^237^ Moreover, iron chelation inhibits adipocyte differentiation without iron overload but can be reversed by transferrin or other iron donors, such as lactoferrin.^140^, ^238^ Deferoxamine's iron chelation improves adipogenesis, reduces oxidative stress, inflammation, and adipocyte hypertrophy, and enhances insulin action.^140^, ^149^, ^239^

Iron overload and protein disorders related to iron metabolism are closely associated with mild traumatic brain injury (mTBI).^240^ This overload can lead to autophagic death and ferroptosis as well as promote tau protein phosphorylation, resulting in nerve fiber tangles and increasing the risk of NDDs such as AD and PD in patients with TBI. In addition, it can damage neural network.^240^

Aβ, a peptide fragment derived from the amyloid precursor protein, aggregates to form insoluble amyloid plaques in the brains of AD patients.^206^ MRI has been used to detect these plaques, primarily due to the presence of iron.^201^, ^206^, ^241^ Moreover, QSM has been conducted to explore the connection between iron deposition and the accumulation of amyloid, using amyloid PET as a diagnostic tool.^38^, ^242^, ^243^ However, experiments have demonstrated that Aβ is diamagnetic and can create strong contrasts on susceptibility maps.^3^, ^206^, ^244^ Researchers have used QSM to monitor iron deposition and Aβ accumulation in an AD transgenic mouse model.^206^ QSM also allows the visualization of individual Aβ plaques and provides a contrast for identifying their deposition and iron. Diamagnetic properties of Aβ have also been observed in brain specimens from AD patients.^180^ In line with these results, Ahmed et al.^245^ used the diamagnetic compartment susceptibility (DCS) from DECOMPOSE‐QSM to track pathological changes in AD. DCS is affected by protein accumulation and demyelination, and a lower absolute value in WM indicates a loss of WM integrity. This study suggests that compartmentalized magnetic susceptibility can provide more holistic information about AD pathogenesis and shows lower DCS values in patients with amnestic dementia than in HCs. Together, these advancements in QSM may help diagnose and track AD‐driven neurodegeneration by non‐invasive assessment of Aβ aggregation and iron deposition.

Furthermore, a recent study using QSM and tau‐PET demonstrated a connection between iron and tau accumulation in 236 patients with amyloid‐β pathology.^202^ This study showed that differences in magnetic susceptibility attributed to increased iron content were consistently associated with tau‐PET signals in regions affected by AD. This study suggests that the accumulation of iron in the brain can affect the development of AD by mediating the relationship between tau‐PET and cortical atrophy. Furthermore, this study found that younger participants (aged ≤65 years) had a stronger association between quantitative susceptibility and tau‐PET. These findings support the idea that iron deposition, tau aggregation, and neurodegeneration are interrelated in AD, and highlight the role of iron dysregulation in the disease process.

Iron plays a critical role in promoting optimal behavioral organization, and its deficiency can impede brain myelination and hinder monoamine metabolism.^246^, ^247^ Changes in brain iron status affect glutamate and γ‐aminobutyric acid homeostasis, causing deficits in memory, learning, motor skills, and emotional and psychological problems.^246^ Iron metabolism influences emotional behavior through region‐specific control, neurotransmitter regulation, oxidative stress responses, and interactions between iron and other metals.^246^, ^247^ In a recent study, the authors examined the relationship between the QSM measurements and AD markers in 421 participants.^38^ The results in line with previous studies showed that older age and cognitive impairment were associated with increased susceptibility in certain brain regions, particularly the deep and inferior gray nuclei.^30^, ^192^, ^216^, ^217^, ^242^, ^243^ Higher susceptibility was also linked to higher levels of amyloid and tau PET, as well as lower cortical GMV in the medial temporal lobe.^38^ These findings suggest that susceptibility to specific brain regions may be indicative of cognitive decline, and the presence of amyloids and tau.

## 4 QUANTITATIVE SUSCEPTIBILITY MAPPING

Conventional MRI cannot accurately show the amount of damage, such as iron deposition, in different components of the central nervous system (CNS), including glia, axons, and myelin.^248^ Magnetic susceptibility is a novel contrast type used in MRI, which differs from other conventional MRI techniques.^229^ Diamagnetic materials exhibit negative susceptibility. Conversely, paramagnetic materials have a positive susceptibility, which means that they are attracted by an applied magnetic field.^27^ The magnetic properties of biological tissues are intricately linked to their molecular composition and microstructure; consequently, they can exhibit diamagnetic or paramagnetic behavior.^27^, ^249^ Most biological substances are slightly diamagnetic, causing small negative susceptibility changes.^250^ However, iron stored in brain tissue and deoxyhemoglobin in venous blood are highly paramagnetic, resulting in a strong magnetic field.^251^

Iron is the main contributor to magnetic susceptibility in various brain structures, particularly the GM.^32^, ^252^ QSM has been used to map the distribution of iron in positive susceptibility (paramagnetic) tissues.^245^, ^253^ Changes in myelin and neurodegeneration‐related accumulation of Aβ or proteins lead to negative susceptibility values in QSM.^245^, ^254^ Despite their colocalization, multiple studies have contradicted the theory that diamagnetic and paramagnetic sources in the brains of patients with AD may affect QSM values. In 2012, Langkammer et al. discovered a strong correlation between the concentration of iron in post‐mortem tissue and the bulk magnetic susceptibility in GM structures. This suggests that iron content is a significant contributor to the bulk of the QSM signal.^32^ Similarly, another study found that there are minimal counteracting effects of paramagnetic and diamagnetic sources when examining differences in susceptibility signatures in AD cases.^255^ These findings support the earlier work of Hallgren and Sourander, who found that iron is the dominant source of magnetic susceptibility in GM.^256^ Therefore, the QSM can be a valuable tool for tracking brain iron deposition.^18^

QSM uses phase signals generated by materials with magnetic susceptibilities different from those of the surrounding tissues.^257^, ^258^ However, phase images cannot be used without additional processing. Phase unwrapping is necessary to handle the dynamic range of “−π to π,” while background field removal is required to address susceptibility differences at tissue‐air boundaries.^3^, ^259^ QSM phase signals are produced by dipole interactions, which occur when materials with varying magnetic susceptibilities are exposed to an external magnetic field.^3^, ^239^ Magnetic susceptibility, which reflects the degree of magnetization, plays a crucial role in this phenomenon.^3^ Phase imaging provides a unique contrast between gray matter, white matter, iron‐laden tissues, venous blood vessels, and other tissues with biologically specific magnetic susceptibilities that differ from those of the background tissues.^18^, ^27^

SWI is a diagnostic technique that capitalizes on the unique magnetic properties of local fields to enhance image contrast and increase the visibility of a wide range of susceptibility sources.^260^, ^261^ SWI serves as a precursor to the concept of QSM.^3^ In SWI, phase images are utilized to generate weighting masks, which are then combined with magnitude images to amplify the susceptibility contrast.^261^ However, SWI is limited by its qualitative nature, as it measures the combined value of the magnitude and homodyne‐filtered phase signals.^3^ The development of QSM has addressed the limitations of accurately assessing magnetic susceptibility in statistical image analyses.^3^, ^262^ This was made possible by solving a complex field‐to‐source inversion problem that separated the local magnetic field from nonlocal contributions. QSM also employs advanced inference techniques to create voxel‐wise maps of mean tissue susceptibility.^18^

**FIGURE 2:**
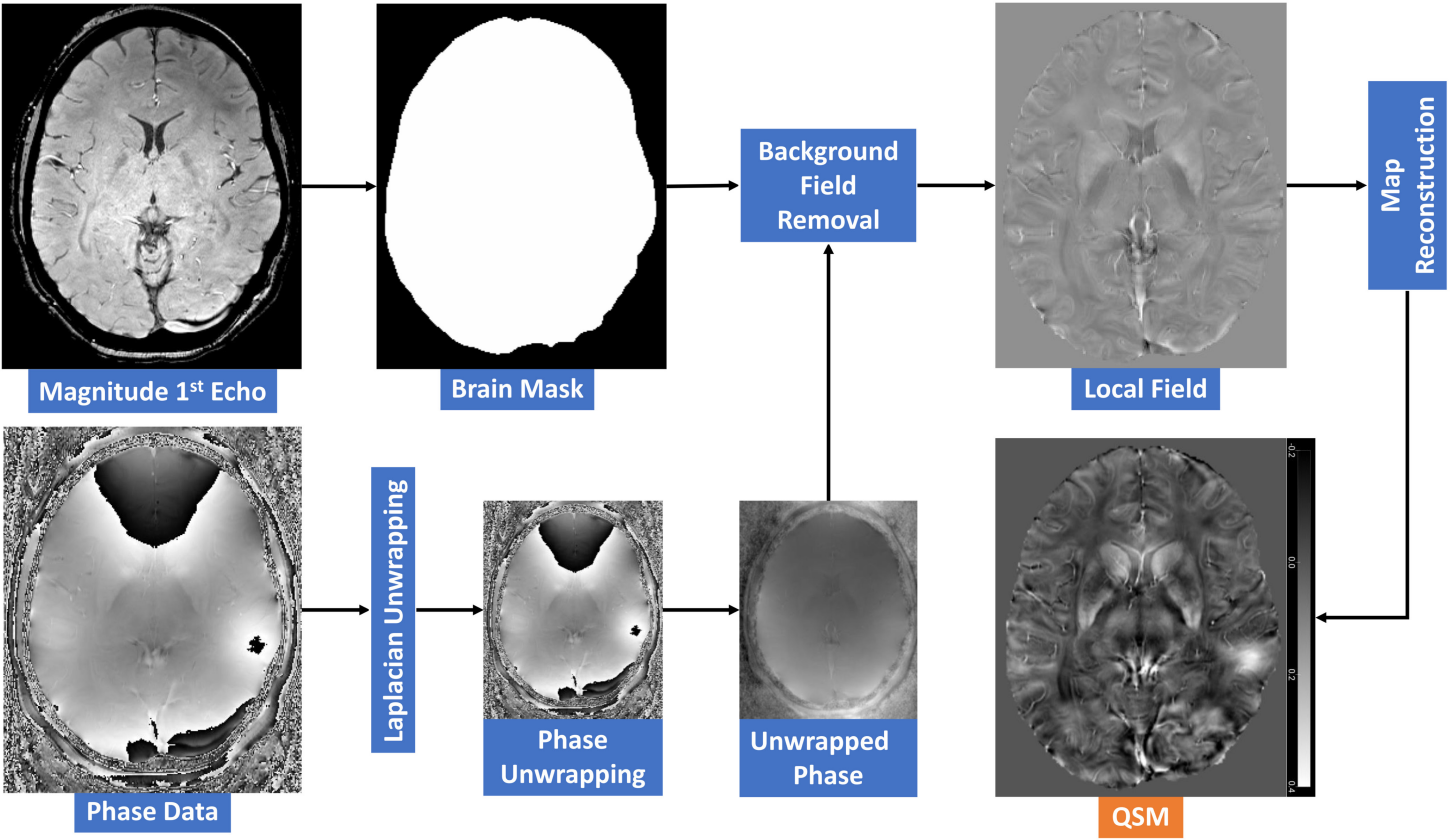
The general post‐processing pipeline of quantitative susceptibility mapping reconstruction steps.

Several recent studies concluded that QSM has the potential to offer valuable pathophysiological insights into the properties of brain tissue and can be used to evaluate the effectiveness of novel therapeutics in clinical settings for early detection of AD.^3^, ^30^, ^38^, ^202^, ^206^, ^216^, ^263^ QSM is a high‐resolution post‐processing MRI technique used to measure local tissue magnetic susceptibility, which is sensitive to metal magnetic susceptibility, such as iron (Figure 2).^3^, ^264^ QSM can accurately measure the magnetic susceptibility of tissues, which indirectly reflects the iron content in the brain. QSM generates a high‐resolution voxel‐wise map of the average magnetic susceptibility of tissues, providing a more precise and informative measurement of brain iron content than conventional imaging techniques.^39^, ^262^ Tissue magnetic susceptibilities can be characterized using QSM, which has various applications, including measuring iron deposition and myelination, quantifying venous oxygen saturation, and assessing blood–brain barrier function.^3^, ^265^ It is worth noting that PET is a high‐accuracy method to detect pathology in AD, but it has some limitations, such as low spatial resolution and exposure to radiation.^38^ Therefore, QSM measurements serve as a complementary technique for overcoming these limitations.^245^

The Electromagnetic Tissue Properties Study Group of the International Society of Magnetic Resonance in Medicine (ISMRM) recommends the use of QSM for clinical brain research.^266^ The group has provided guidelines for the acquisition, processing, analysis, and publication of QSM data. To obtain accurate results, it is recommended to use a monopolar 3D multi‐echo gradient echo sequence and save the phase images in the DICOM format. A brain mask with phase quality‐based masking should be utilized to remove background fields, and techniques such as Sophisticated Harmonic Artifact Reduction for Phase data (SHARP) or projection onto Dipole Fields (PDF) should be applied to remove any remaining background fields within the mask. Finally, the susceptibility values should be measured relative to the reference region to ensure the accuracy of the results.

## 5 EMERGING ALTERATIONS IN THE PRECUNEUS/POSTERIOR PARIETAL CORTEX

### 5.1 Magnetic susceptibility patterns

A preliminary QSM study suggested significant differences in magnetic susceptibility between patients with AD and matched HCs in the deep brain nuclei, especially in the putamen, and the posterior GM and WM regions in both regional and whole‐brain cross‐sectional comparisons.^216^ In the cortical areas, the greatest increase in susceptibility values with age occurred in regions responsible for cognitive processing, such as the precuneus.^15^ The accumulation of iron in the basal ganglia showed a linear trend, whereas iron accumulation in the precuneus followed a quadratic or exponential pattern.^15^ Notably, a phase‐shift study revealed that there is a positive correlation between iron concentration in the parietal cortex and the severity of cognitive impairment in AD patients.^267^ This finding suggests that iron concentration can be utilized as a biomarker to assess the progression of AD. This is aligned with a recent study that suggests that brain iron accumulation (BIA) occurs in areas such as the precuneus in AD.^26^

Kim et al.^30^ investigated the susceptibility of the precuneus in differentiating early‐stage AD and aMCI from HCs. Their findings indicated that the area under the curve (AUC) was 0.85, which suggests a high level of accuracy in distinguishing AD patients from HCs. QSM values increased in the aMCI and AD groups, whereas GMVs decreased. The QSM was more effective in distinguishing individuals with aMCI from cognitively normal individuals in specific brain regions. Changes in the QSM values of these lesions in AD and amyloid‐beta‐cingulate gyrus may indicate the contribution of both amyloid accumulation and NFTs.^30^, ^268^ Susceptibility alterations start in the aMCI stage, making QSM values in the precuneus and allocortex better than GMV values for investigating early brain changes.^30^ According to this study, QSM has the potential to serve as a valuable supplementary imaging technique for the timely detection of AD, especially in areas where Aβ and iron accumulate.

Another study that used high‐resolution 7 Tesla T2*‐w imaging found an increased peak‐to‐peak phase shift in all lobar regions of patients with early onset AD (EOAD) than in patients with late‐onset AD (LOAD).^269^ The regional mean phase contrast in patients with EOAD was higher in the parietal subregions, especially in the precuneus. This indicates an increased accumulation of iron, which may be related to higher amyloid deposition in specific cortical regions than in patients with LOAD.

Taken together, the QSM studies revealed significant differences in magnetic susceptibility between patients with AD and HCs in the posterior GM and WM regions, suggesting greater iron accumulation in the dorsal nervous system, including the precuneus. Thus, QSM values can better distinguish early AD changes from GMV values, and may be a useful tool for early AD diagnosis.

### 5.2 Phase difference‐enhanced (PADRE) imaging

Phase difference‐enhanced (PADRE) imaging was expanded to enhance the phase differences between the target and surrounding tissues, allowing visualization of the target tissue.^270^ This technique has been used to differentiate the pathologies of CNS diseases and detect early pathological changes in certain NDDs.^271^, ^272^ Some studies based on PADRE imaging have been conducted. One study found a significant positive correlation between Amyloid‐β‐enhancing PADRE imaging and the standardized uptake value ratio (SUVR) of amyloid PET in the precuneus and cuneus, suggesting its potential for predicting the SUVR of amyloid PET.^271^ Another study indicated the feasibility of using PADRE imaging at 3T to differentiate between AD patients and control subjects.^273^ In a visual evaluation of PADRE images enhanced for amyloid‐β, areas of hypointensity were more pronounced within the cerebral cortex of AD patients than in HCs.^273^

In general, initial studies have suggested that PADRE imaging correlates with amyloid PET for detecting AD pathology and may distinguish AD from HCs, demonstrating its potential as a noninvasive alternative to PET for AD diagnosis and prognosis.

### 5.3 Cerebral metabolic rate of oxygen consumption and oxygen extraction fraction

Blood vessels in the brain play a crucial role in AD.^274^ Monitoring central venous oxygenation can serve as a new way to study how the brain's blood flow is affected, helping us understand vascular disorders that impact oxygen supply.^3^, ^275^ Physiological variables such as CBF, oxygen extraction fraction (OEF), and cerebral metabolic rate of oxygen consumption (CMRO2) are crucial for understanding brain oxygen utilization, blood supply, and energy consumption (Equation 1).^40^ These parameters are potential biomarkers of AD, with most arterial spin labeling (ASL) studies showing a decrease in CBF.^40^, ^41^, ^42^, ^43^, ^44^, ^45^, ^46^, ^47^, ^48^, ^49^, ^50^, ^51^, ^52^ Distinguishing between vascular cognitive impairment and AD is challenging because of overlapping underlying causes. However, changes in brain OEF can help differentiate between the two.^3^, ^276^

(1) $$\text{CMRO}2=\mathrm{CBF}\times \mathrm{OEF}\times \left[H\right]a$$

The CMRO2 was measured in μmol/100 g/min. CBF was measured in mL/min/100 g. The OEF was expressed as a percentage. [H]a = 7.377 μmol/mL is the molar concentration of oxygenated heme in an arteriole with hematocrit (Hct) = 0.357.^40^

A recent study aimed to evaluate the whole‐brain patterns of OEF, CBF, and CMRO2 perturbation in AD.^40^ The researchers used the cluster analysis of time evolution and tissue composition (CCTV) algorithm, which combines QSM and quantitative blood oxygen level‐dependent (BOLD) models (QQ) to calculate OEF.^40^ The results showed that CMRO2 and CBF values were significantly decreased in AD patients, predominantly in the bilateral precuneus gyrus, angular gyrus, and parietal–temporal regions. Regional analysis revealed decreased CBF and CMRO2 in the bilateral caudal and left rostral hippocampi. This study suggests that CMRO2 mapping could be a potential biomarker for AD and a useful tool for monitoring cognitive impairment. On the other hand, QSM is used to measure venous oxygen saturation, allowing the calculation of CMRO2 and OEF.^40^, ^277^, ^278^

## 6 CONCLUSIONS AND SUMMARY

Substantial neuroimaging evidence indicates that the precuneus/posterior parietal cortex is vulnerable in early AD pathogenesis. Emerging findings using QSM demonstrate greater iron deposition compared to HCs, even at the MCI stage. Our evidence is supported by previous MR neuroimaging findings such as hypoperfusion, atrophy, cortical thinning, and connectivity disruption in the precuneus. Additionally, metabolic disorders such as obesity, high VAT, diabetes, and NAFLD are associated with this imaging evidence. Iron overload can promote several pathogenic processes implicated in AD, including amyloid aggregation, lipid peroxidation, tau phosphorylation, and neuronal death pathways such as ferroptosis. Higher magnetic susceptibility is also associated with metabolic risk factors such as obesity, IR, and vascular pathology.

Current research on iron accumulation and AD in the precuneus using QSM is emerging and promising, but there are limitations to address in future studies. Standardized QSM implementation processing is necessary to more accurately distinguish AD from MCI or HCs. Moreover, ongoing longitudinal studies are warranted to determine how iron accumulation measured by QSM translates into disease progression and cognitive decline.

Together, these findings suggest that iron accumulation, measurable through QSM, may accelerate pathogenesis in a sensitive hub region, contributing to downstream atrophy and cognitive deficits. As an early non‐invasive neuroimaging biomarker of iron burden and oxidative stress, QSM holds promise for improving early diagnosis, tracking disease stages, and monitoring therapeutic responses in AD and related dementias

## AUTHOR CONTRIBUTIONS

SM: conceptualization, methodology/study design, software, data curation, writing – original draft preparation, visualization, investigation, validation, formal analysis, writing – reviewing, and editing. SG: conceptualization, methodology/study design, software, data curation, writing – original draft preparation, visualization, investigation, validation, formal analysis, writing – reviewing, and editing. FF: supervision, validation, writing – reviewing, and editing.

## FUNDING INFORMATION

This research did not receive any specific grants from funding agencies in the public, commercial, or not‐for‐profit sectors.

## CONFLICT OF INTEREST STATEMENT

The authors declare that they have no conflicts of interest.

## ETHICAL APPROVAL

None of the human or animal subjects were included in this study.

## Data Availability

The data supporting the findings of this study are available upon request from the corresponding author.
